# *TET2* mutations and clonal dynamics

**DOI:** 10.18632/oncotarget.26779

**Published:** 2019-03-12

**Authors:** Ashwin Kishtagari, Babal K. Jha, Jaroslaw P. Maciejewski

**Affiliations:** Jaroslaw P. Maciejewski: Department of Translational Hematology and Oncology Research, Lerner Research Institute, Cleveland Clinic, Cleveland, OH, USA; Leukemia Program, Department of Hematology and Medical Oncology, Taussig Cancer Institute, Cleveland Clinic, Cleveland, OH, USA

**Keywords:** clonal hierarchy, TET2, mutations

The TET α-ketoglutarate (α-KG)-dependent DNA dioxygenases (*TET1-3*) catalyze in the presence of Fe^2+^ and ascorbic acid the successive oxidation of 5-methylcytosine (5mC) to 5-hydroxymethylcytosine (5hmC) and other oxidation products down to 5-carboxylcytosine [1]. During replication, 5hmC is not recognized by the methyltransferase and thus, among many consequences of mutations, it is believed that indirect/passive demethylation is a main function of TET proteins. Expression of TET genes are highly regulated to create tissue-specific patterns. *TET2* is most abundant in hematopoietic system especially along monocytic and lymphoid differentiation [2]. *TET2* inactivation (but not TET1 or TET3) through loss-of-function/hypomorphic mutations (*TET*^*MT*^) is a common clonal event in myeloid and T-lymphoid neoplasms [3, 4]. However, the function of TET enzymes can also be affected by acetylation or phosphorylation and thus it is not clear what proportion of protein is functionally active. Somatic *TET2*^*MT*^ are encountered in 25-30% of patients with myelodysplastic syndromes (MDS), myeloproliferative neoplasms (MPN), overlap syndromes and AML, with 50-60% chronic myelomonocytic leukemias (CMML) harboring mono or biallelic *TET*^*MT*^ [3-6]. On the cellular level, *TET2* is involved in regulation of hematopoietic stem cell (HSC) self-renewal, and myeloid lineage commitment of progenitor cells and stem cells (HSPCs). Conditional loss of *TET2* in mice leads to protracted expansion of HSPCs with skewed differentiation towards monocytic progenitors, splenomegaly and extramedullary hematopoiesis with an overall mild phenotype [7].

**Figure 1:**
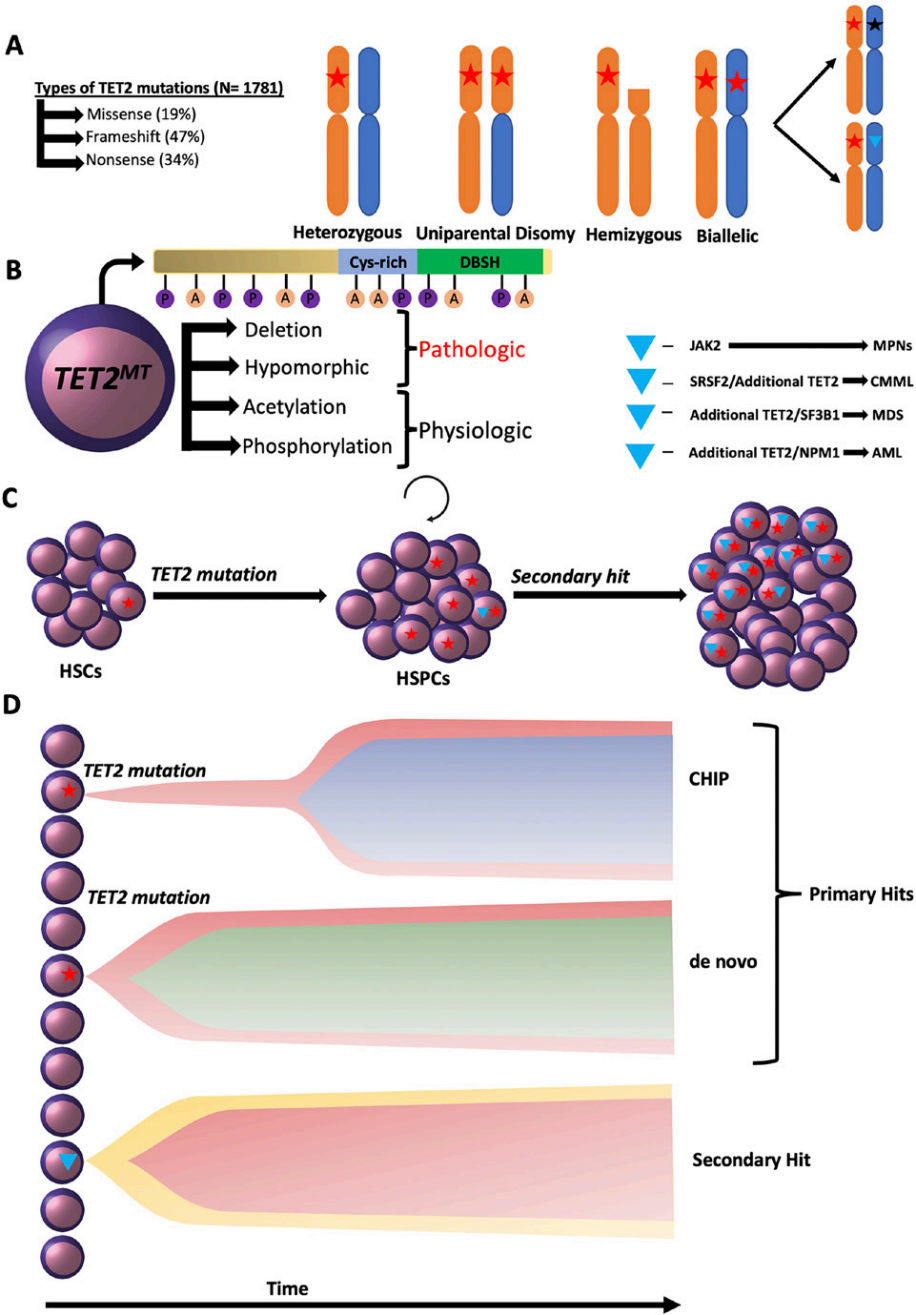
**A.** Distribution and configuration across the spectrum of TET2 mutations. **B.** Posttranslational modifications of TET2 protein. **C.** & **D.** Clonal hierarchy of TET2 mutations.

Are TET2 mutations driver lesions of leukemogenesis? Leukemogenesis is a multistep process creating combinatorial and hierarchical diversity. Herein the majority *TET2*^*MT*^ are likely ancestral events in leukemogenesis [5], although they can also occur as secondary or early hits following other common mutations such as *ASXL1* or SRSF2 [5]. Consistent with such a notion, *TET2*^*MT*^ have been detected in seemingly asymptomatic older controls, also referred as to having clonal hematopoiesis of indeterminate potential (CHIP), which is associated with an increased risk of hematologic malignancies (albeit a long latency and a low penetrance) [8]. While clonal evolution may be related to increased fitness of *TET2*^*MT*^ stem/progenitor cells, the actual mechanism is not entirely defined. *TET2*^*MT*^ may predispose to additional somatic events (2^nd^ *TET2, SRSF2, JAK2, SF3B1,* and *NPM1*), which are required to drive leukemic transformation. Thus, it is possible that *TET2*^*MT*^ convey a clonally restricted mutator phenotype. Indeed, *TET2*^*MT*^ patients and knock-out (KO) murine strains acquire more somatic mutations. *TET2*^*WT*^ hypermutagenicity in murine *tet2*^*K*D^ HSCs/HSPCs led to the accumulation of secondary mutations [9]. However, the absence of *TET2*^*MT*^ in childhood and increasing *TET2*^*MT*^ incidence with aging (up to 80% of MDS cases in the 8^th^ decade of life harbor *TET2*^*MT*^) suggest that changes related to aging may form the environment, which either promotes *TET2*^*MT*^ clonal expansion or increases the odds of acquisition of this hit. Such changes may involve corroborating pathways, but also the direct modification of *TET2, e.g*., by phosphorylation or acetylation (Figure 1B). Given the low penetrance, long latency, their lack of impact on outcomes including risk of progression or incremental pathogenicity of biallelic inactivation of *TET2*^*MT*^ and deletions, they should not be considered typical driver mutations.

Prognostic and morphologic impact of TET2 mutations? One explanation for modest effect of *TET2*^*MT*^ on progression or survival is the heterogeneous topology of *TET2*^*MT*^, their configuration, sub-clonal context, and the co-occurring mutations [2, 5]. However, it is also plausible that TET2 activity is modulated by protein modifications, which in effect may further worsen or alleviate the effects of heterozygous mutations. In our recent publication of 1205 cases with 1781 *TET2* mutations the diversity was indeed tremendous including homo/hemizygous and biallelic lesions, frameshift (47%), non-sense (34%), and missense (19%) in various positions within the gene (Figure 1A). Hierarchical analysis suggests that most *TET2*^*MT*^ represent phenotype-neutral ubiquitous ancestral hits, which seem to create a leukemogenic predisposition (mutator phenotype) rather than leukemic drive. However, clonal rank as an ancestral hit conveys predilection for a limited number of secondary hits which in turn may shape morphologic features or even outcome. For instance, following a founder *TET2* lesion, secondary *JAK2* hit determines myeloproliferative features, SRSF2 and additional *TET2*^*MT*^ determine the myelomonocytic phenotype, and *DNMT3A, U2AF1* and *SF3B1* shape the morphology along the dysplastic path (Figure 1B&1C). In contrast, the presence of *TET2*^*MT*^ prevents the evolution of *IDH1/2* mutant clones to evolve and *vice versa,* strongly suggesting that complete inhibition of α-KG dependent dioxygenase activity by the inhibitory oncometabolite 2 hydroxyglutarate is deleterious to *TET2*^*MT*^ cells.

In conclusion, clarification of the mechanistic consequences of *TET2*^*MT*^ on biochemical and cellular levels will require better understanding of the genotype-phenotype associations. The ubiquitous nature of *TET2*^*MT*^ in aging marrow, long latency and incomplete penetrance suggest that they do not promote leukemogenesis in the manner one would expect from canonical driver hits.
